# Supplementary material for: Comprehensive characterization of the antibody responses to SARS-CoV-2 Spike protein after infection and/or vaccination
Source: bioRxiv. 2021 Oct 5:2021.10.05.463210. Preprint. [Version 1] doi: 10.1101/2021.10.05.463210 (PMC8509098; doi:10.1101/2021.10.05.463210)
Supplement: 1 [file NIHPP2021.10.05.463210V1-supplement-1.pdf]

bioRxiv preprint doi: https://doi.org/10.1101/2021.10.05.463210; this version posted October 7, 2021. The copyright holder for this preprint (which was not certified by peer review) is the author/funder, who has granted bioRxiv a license to display the preprint in perpetuity. It is made available under aCC-BY 4.0 International license.

SUPPLEMENTAL FIGURES

Supplemental Figure 1

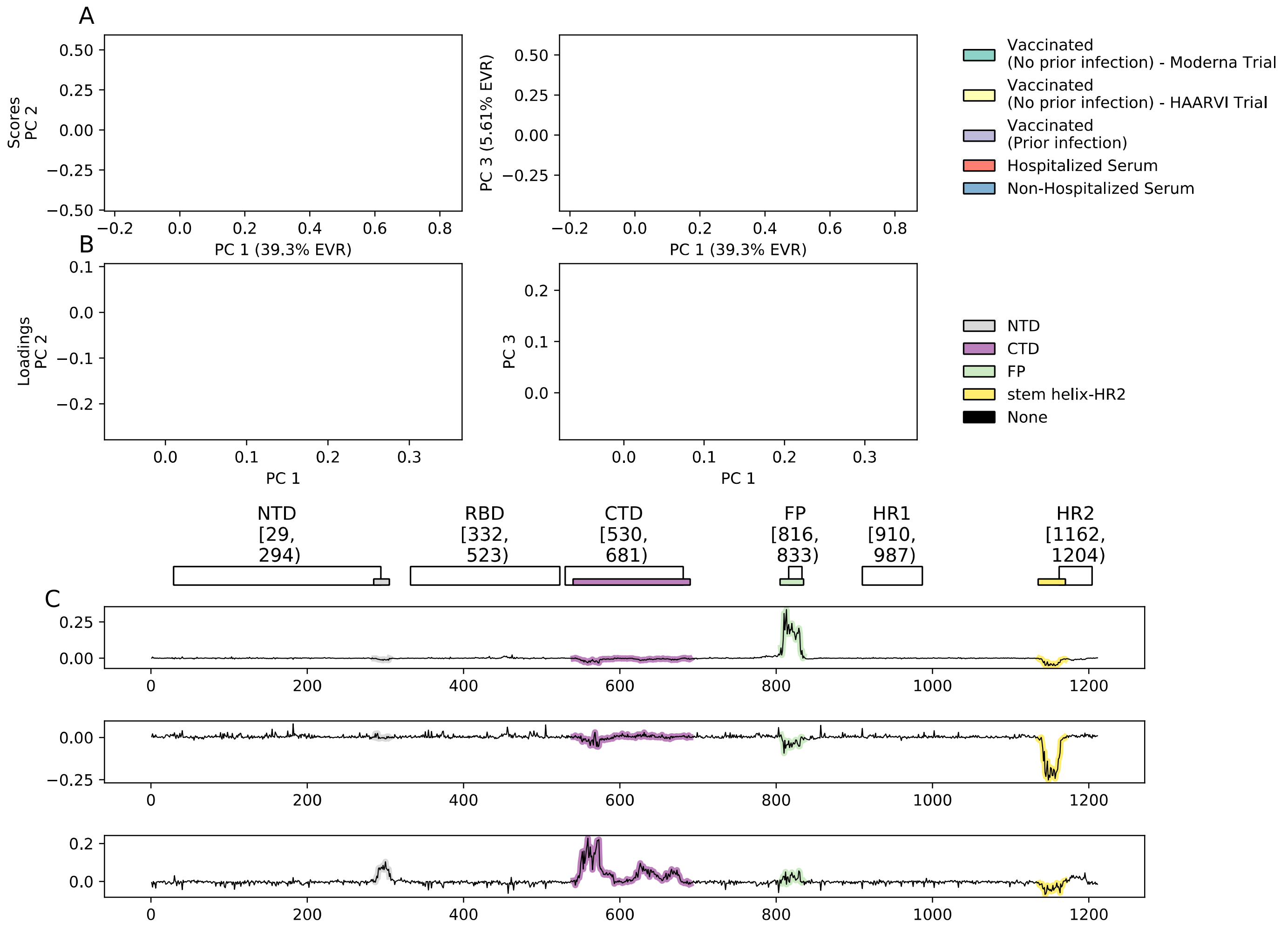

SUPPLEMENTAL FIGURE 1: **Principal Component Analysis on wild-type enrichment features of all samples** (A) Scatterplot depicting the unit scaled sample "scores" represented by the columns to visualize sample relationship in principal component space. Colors represent the group which each sample belongs to. (B) Vector plots showing the component loadings, scaled by the square root of the respective eigenvalues in the eigen-decomposition. Colors represent the genomic location of each component loading score. (C) Line plots showing the first three principal axes/directions in feature space, plotted as a function of the wild-type peptide feature location on Spike.

bioRxiv preprint doi: https://doi.org/10.1101/2021.10.05.463210; this version posted October 7, 2021. The copyright holder for this preprint (which was not certified by peer review) is the author/funder, who has granted bioRxiv a license to display the preprint in perpetuity. It is made available under aCC-BY 4.0 International license.

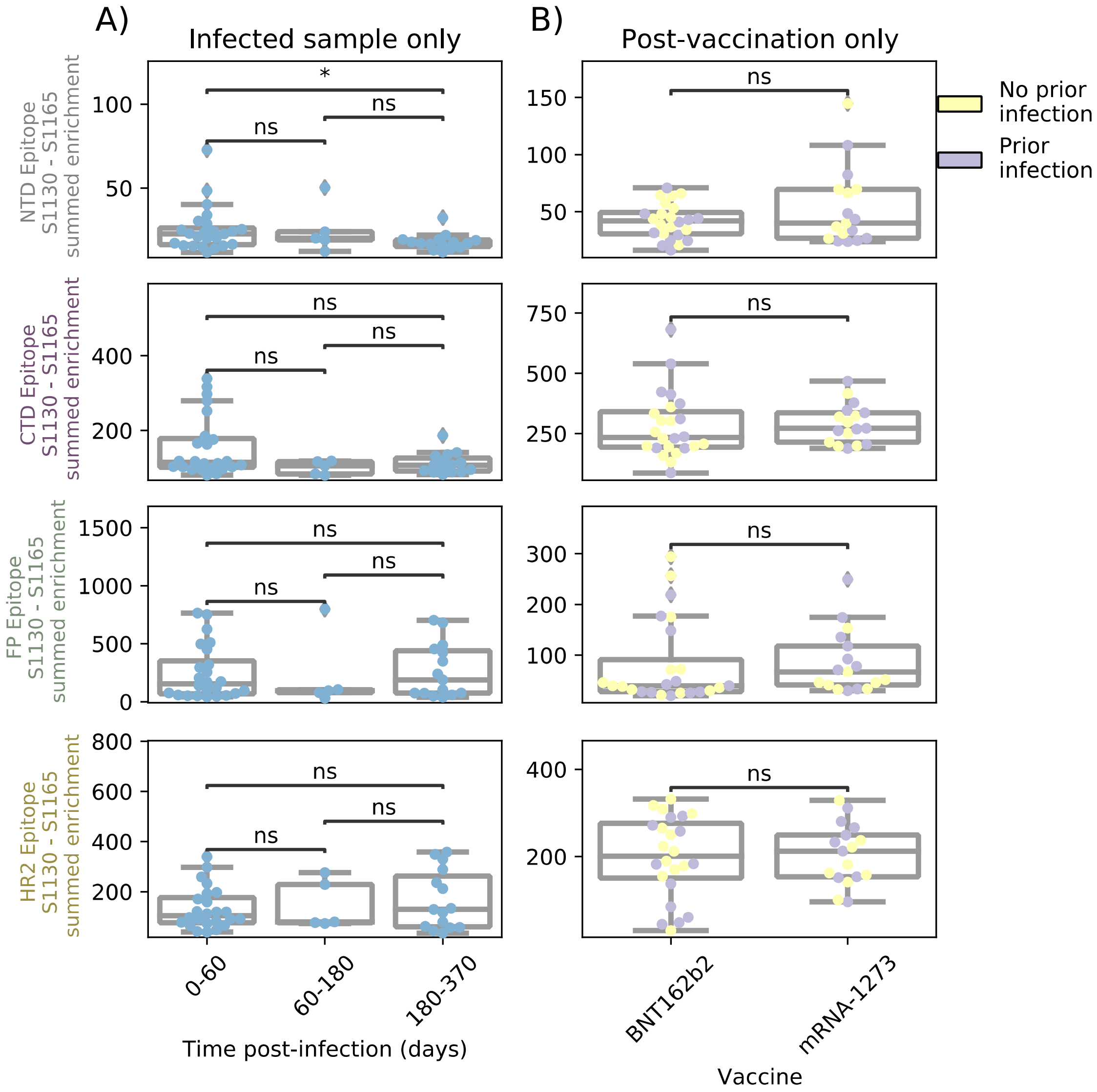

SUPPLEMENTAL FIGURE 2: **Comparison of epitope binding for HAARVI subgroups.** Boxplots of summed wild-type enrichment within epitope binding regions for samples grouped by (A) timepoint post symptom onset or (B) vaccine type (Pfizer/BioNTech BNT162b2 or Moderna mRNA-1273). Results of a Mann-Whitney test between the groups are shown. P-values were adjusted for multiple testing using Bonferroni correction. * indicates p<0.05, ns means “not significant”.

bioRxiv preprint doi: https://doi.org/10.1101/2021.10.05.463210; this version posted October 7, 2021. The copyright holder for this preprint (which was not certified by peer review) is the author/funder, who has granted bioRxiv a license to display the preprint in perpetuity. It is made available under aCC-BY 4.0 International license.

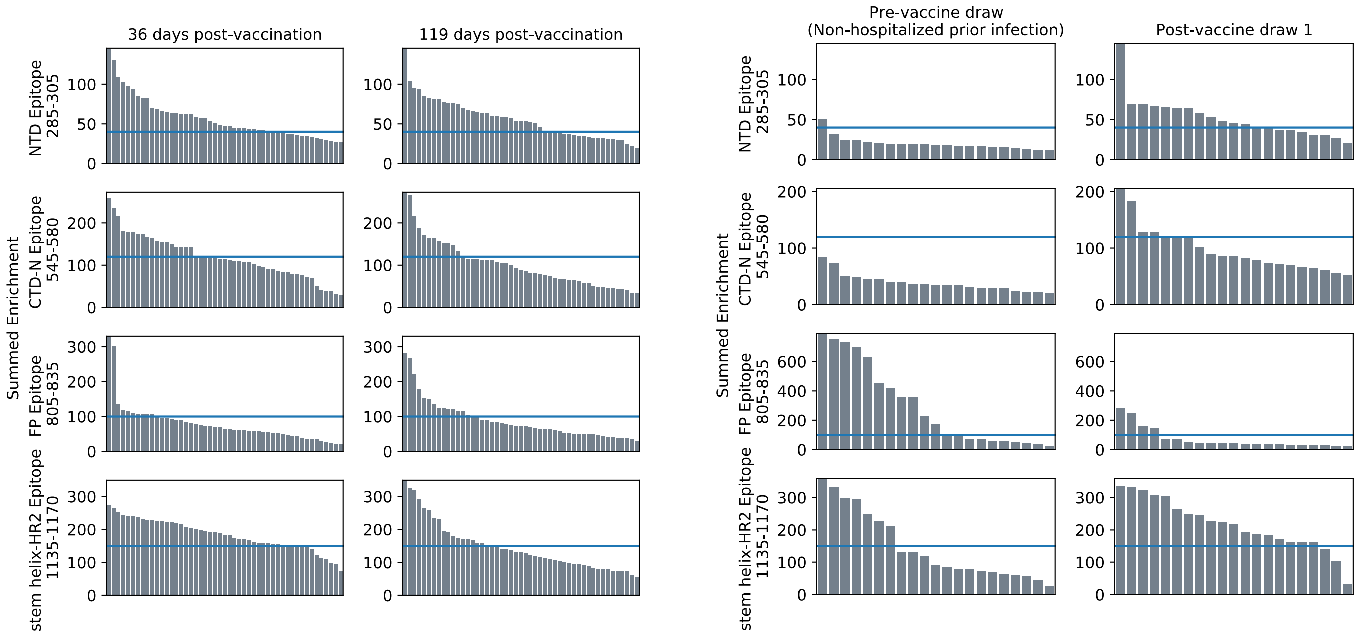

SUPPLEMENTAL FIGURE 3: **Thresholding of total epitope binding within major epitope regions.** Histogram showing the summed enrichment values within each epitope region for every sample in the Moderna Trial Cohort (left two panels) or HAARVI Cohort (right two panels). Blue line delineates the threshold chosen for each epitope region. Samples above the line were included in the escape profile analyses.
